# Retraction: Is insulin resistance the cause of fibromyalgia? A preliminary report

**DOI:** 10.1371/journal.pone.0226174

**Published:** 2019-12-02

**Authors:** The PLOS ONE Editors

After publication of [1], concerns were raised about the lack of prospective ethics approval for the study, whether the study design was sufficiently rigorous to address the study aims, and whether the conclusions were adequately supported. In light of these concerns, we reassessed the article and obtained input on the specific issues from an Academic Editor and a member of *PLOS ONE*’s Human Research Advisory Group.

During the pre-publication review process, when queried about ethics oversight, the authors said that this study was exempt from ethics review because it involved a retrospective review of anonymized medical records and no treatment was prospectively administered for the sake of this research. Prior to publication and in response to journal queries, the authors provided a document dated after completion of the study confirming that an independent ethics review board had reviewed the study and deemed it exempt from the requirement for ethics review according to U.S. regulation 45 CFR §46.104 Category #4. Based on the information provided prior to publication, the *PLOS ONE* Editors considered the review board’s exemption decision as sufficient to address the journal’s ethics requirement, even though our policy states that the review board should have been consulted prior to the study.

In our post-publication discussions with the authors it came to light that one or more author(s) in principle had access to identifying participant information and that the study involved patients from the authors’ clinical practice. The authors believed that permission to access patient records was covered by the established patient-doctor relationship. Per *PLOS ONE* standards an IRB needs to be consulted before conducting any study in which identifiable patient records are accessed for the purpose of research. An Academic Editor and a member of *PLOS ONE*’s Human Research Advisory Group consulted post-publication both advised that in their assessment, an institutional review board or equivalent ethics committee ought to have been consulted prior to the retrospective study given the authors’ ability to access patient-identifying information. Based on our further editorial assessment of this matter, the information that came to light about the study post-publication, and advice received in our external consultations, the *PLOS ONE* Editors have determined that the study did not meet the journal’s requirements, although per the independent review board’s decision the study may have met local requirements.

The Academic Editor consulted in the post-publication assessment also raised concerns that the study did not include appropriate controls. HbA1c values in study participants were compared to mean HbA1c values in published datasets, and the Academic Editor advised that the study ought to have included age-, sex-, and ethnicity-matched controls from the same clinic population as those who received metformin. This information was not requested during the pre-publication review process, and the authors commented that this could in principle have been addressed prior to publication or post-publication through inclusion of additional data and revision of the article.

Owing to these concerns, the *PLOS ONE* Editors retract this article. We regret that these issues were not identified and fully addressed during the article’s pre-publication review.

MAP, CRA, NHG, FA, XF, MAS, AMT did not agree with retraction. LM did not respond or could not be reached.
